# Prickle3 synergizes with Wtip to regulate basal body organization and cilia growth

**DOI:** 10.1038/srep24104

**Published:** 2016-04-11

**Authors:** Chih-Wen Chu, Olga Ossipova, Andriani Ioannou, Sergei Y. Sokol

**Affiliations:** 1 Department of Developmental and Regenerative Biology, Icahn School of Medicine at Mount Sinai, New York, NY 10029, USA

## Abstract

PCP proteins maintain planar polarity in many epithelial tissues and have been implicated in cilia development in vertebrate embryos. In this study we examine Prickle3 (Pk3), a vertebrate homologue of *Drosophila* Prickle, in *Xenopus* gastrocoel roof plate (GRP). GRP is a tissue equivalent to the mouse node, in which cilia-generated flow promotes left-right patterning. We show that Pk3 is enriched at the basal body of GRP cells but is recruited by Vangl2 to anterior cell borders. Interference with Pk3 function disrupted the anterior polarization of endogenous Vangl2 and the posterior localization of cilia in GRP cells, demonstrating its role in PCP. Strikingly, in cells with reduced Pk3 activity, cilia growth was inhibited and γ-tubulin and Nedd1 no longer associated with the basal body, suggesting that Pk3 has a novel function in basal body organization. Mechanistically, this function of Pk3 may involve Wilms tumor protein 1-interacting protein (Wtip), which physically associates with and cooperates with Pk3 to regulate ciliogenesis. We propose that, in addition to cell polarity, PCP components control basal body organization and function.

During development, embryonic cells often become oriented in the plane of the tissue, causing visibly polarized patterns, such as in the *Drosophila* cuticle or mammalian hair. This phenomenon is known as planar cell polarity (PCP). Studies of *Drosophila* epithelia demonstrated that PCP is maintained largely by molecular interactions that lead to mutually exclusive distribution of two ‘core’ protein complexes, Frizzled (Fz)-Dishevelled (Dvl) and Van Gogh (Vang)-Prickle (Pk) to opposite sides of an epithelial cell^1^,^2^. In vertebrate embryos, planar cell polarity signaling has been documented in several tissues, such as the cochlea, the skin, and the neural plate^3^,^4^,^5^,^6^,^7^,^8^,^9^,^10^. Furthermore, loss-of-function studies established a requirement of vertebrate PCP components in diverse cell behaviors including mediolateral and radial cell intercalations, apical constriction and cell migration^8^,^11^,^12^,^13^,^14^,^15^,^16^. This evidence demonstrates the expanded roles of vertebrate PCP proteins in multiple developmental processes.

Besides morphogenetic events, PCP components are involved in other processes associated with cell polarity, such as asymmetric cell division^17^,^18^ or left-right patterning^19^,^20^,^21^,^22^. Several core PCP proteins are enriched at the centrosome and/or basal body^23^,^24^,^25^,^26^,^27^,^28^, but the functional significance of this localization is not understood. Consistent with a possible function at the basal body, core PCP proteins have been shown to control cilia development and function^19^,^20^,^21^,^22^,^23^,^29^,^30^,^31^. Nevertheless, the role of PCP proteins in basal body organization largely remains to be investigated.

*Drosophila* Prickle is a cytoplasmic LIM domain protein, which physically associates with Vang and functions in PCP establishment^32^,^33^. The existence of four vertebrate *prickle* gene homologues suggests that they may have diverged from each other and acquired unique functions, as reported for *celsr/flamingo* genes^34^. Consistent with this possibility, the vertebrate Prickle family has been implicated in a number of developmental processes, including convergent extension movements, apicobasal and planar cell polarity^35^,^36^,^37^,^38^,^39^. Moreover, interference with Pk1 and Pk2 functions resulted in defective cilia growth and morphology^40^,^41^,^42^,^43^. Prickle3 (Pk3), also known as LMO6, is a largely unstudied family member that has been implicated in radial intercalation of multi-ciliated cells in *Xenopus* ectoderm^13^. In mammalian cells, Pk3 was reported to localize to mitotic spindle poles and centrioles^44^,^45^, suggesting that it may be involved in centrosome function.

To study roles of Pk3 in PCP and basal body/centrosome function, we examined *Xenopus* gastrocoel roof plate (GRP). GRP is homologous to mouse posterior notochord or zebrafish Kupffer’s vesicle and represents the posterior-most part of the archenteron (Fig. 1A)^46^,^47^,^48^,^49^,^50^. In this tissue, motile cilia generate unilateral fluid flow critical for left-right patterning, which is controlled by PCP signaling^51^. We report that GFP-Pk3 is enriched at the basal body of GRP cells. Overexpression of Vangl2 leads to the recruitment of Pk3 to anterior cell borders in GRP cells, consistent with endogenous Vangl2 distribution. Interference with Pk3 activity in GRP cells suppressed anterior Vangl2 polarization, posterior cilia positioning and cilia growth. In addition to the roles of Pk3 in PCP and cilia formation, we find that Pk3 is required for γ-tubulin and Nedd1 recruitment to the basal body, indicating a new function in basal body organization. We show that this function may be mediated through the association and functional synergy of Pk3 with Wilm’s tumor protein**-**1-interacting protein (Wtip), a vertebrate-specific LIM domain protein.

## Results

### Pk3 is localized to the basal body of GRP cells, but becomes polarized to the anterior cell boundary in the presence of Vangl2

**Figure 1:**
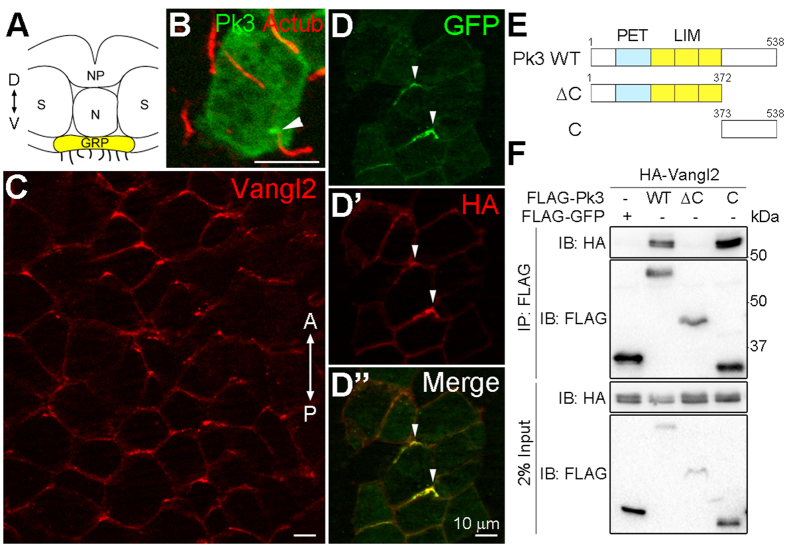
Localization of Pk3 and Vangl2 in the gastrocoel roof plate (GRP). (**A**) Schematic neurula embryo section illustrating the position of the GRP (yellow). The dorsal (D)- ventral (V) axis is indicated. NP, neural plate; N, notochord; S, somites. (**B**) Enrichment of GFP-Pk3 at the base of cilium in GRP cells (arrowhead). Embryos were injected with GFP-Pk3 RNA (0.25 ng). Protein localization is detected by GFP fluorescence in stage 15 GRP explants. Cilium is stained by anti-acetylated α-tubulin (Ac-tub) antibody. (**C**) *En face* immunostaining of endogenous Vangl2 in the GRP of stage 15 embryos. The anterior (A) - posterior (P) axis is indicated. Note the accumulation of Vangl2 at anterior cell boundaries. There is no staining in the absence of primary antibody (data not shown). (**D–D**”) GRP cells coexpressing GFP-Pk3 and HA-Vangl2 (150 pg of RNA each) at stage 15. Arrowheads mark anterior cell borders. (**E**) Scheme of Pk3 constructs. (**F**) Physical interaction between Pk3 and Vangl2 in transfected HEK293T cells. The indicated FLAG-Pk3 proteins were pulled down using anti-FLAG-agarose beads, and co-precipitation of HA-Vangl2 was examined. Protein levels are shown by immunoblotting with anti-FLAG and anti-HA antibodies.

We have recently shown that RNA encoding Pk3 is present in the early *Xenopus* embryo^13^ and decided to assess protein subcellular localization. The GRP tissue allows the examination of both basal body/cilia development and planar polarity, which is manifested by posterior positioning of cilia in each cell^22^,^49^,^52^. In GRP cells, GFP-Pk3 was present throughout the cytoplasm and enriched at the base of the cilium (Fig. 1A,B). By contrast, we observed that endogenous Vangl2 was enriched at anterior cell edges (Fig. 1C), virtually identical to its distribution in neural plate cells^6^.

Since Prickle and Vang are known to physically interact^33^,^53^, we wanted to test whether Vangl2 can promote Pk3 polarization at the cell cortex. Indeed, in the presence of Vangl2, Pk3 was recruited to the anterior cell boundary in mosaically-expressing GRP cells (Fig. 1D–D’’), consistent with the anterior distribution of endogenous Vangl2. This observation suggests that Pk3 cortical localization may be regulated by Vangl2. We next evaluated physical association between Pk3 and Vangl2 in transfected human embryonic kidney 293T (HEK293T) cells, in order to identify the Pk3 domain responsible for this interaction. Although *Drosophila* Pk binds Vang through its C-terminal tail^33^, the C-termini of Pk3 and Pk are highly divergent, with less than 11% amino acid identity (data not shown). We, therefore, generated the C-terminal Pk3 construct (Pk3C) and the one that lacks the C-terminus (Pk3ΔC) to test their association with Vangl2 (Fig. 1E). Immunoprecipitation analysis showed that HA-Vangl2 was efficiently pulled down with FLAG-tagged Pk3 and Pk3C but not Pk3∆C (Fig. 1F), indicating that the Vangl2-binding domain is located at the C terminus of Pk3. Together, these observations demonstrate that Pk3 accumulates at the basal body of GRP cells and can be recruited to the anterior cell boundary by Vangl2.

### Interference with Pk3 activity reveals its roles in cell polarity and ciliogenesis

**Figure 2:**
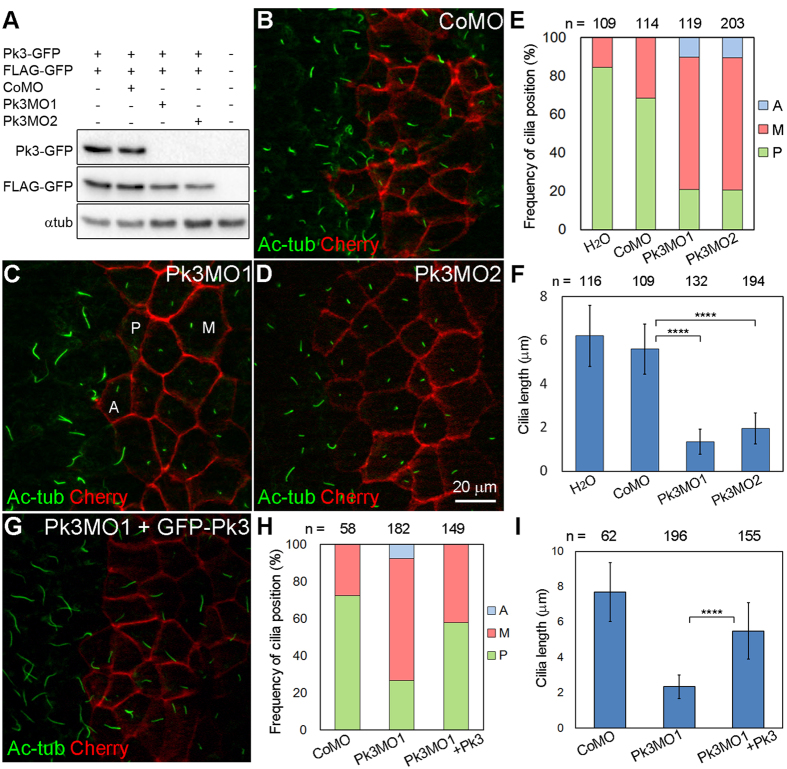
Pk3 is required for the posterior localization and growth of GRP cilia. (**A**) Efficiency of the Pk3 knockdown. Embryos were injected with Pk3-GFP RNA (2 ng) that contains MO target sites, control FLAG-GFP RNA (0.1 ng), control MO (CoMO, 80 ng), Pk3MO1 (15 ng), or Pk3MO2 (80 ng) as indicated. Embryo lysates obtained at stage 11 were immunoblotted with anti-GFP antibody. α-tubulin (αtub) is a control for loading. (**B–I**) Effects of Pk3 depletion (**B–F**) and rescue (**G–I**) on GRP cilia position and length. Embryos were injected at the 4–8 cell stage with CoMO (**B**), Pk3MO1 (**C**), Pk3MO2 (**D**) or Pk3MO1 plus GFP-Pk3 RNA (10 pg, **G**). GRP explants were prepared from stage 17 embryos and stained with anti-acetylated α-tubulin (Ac-tub) antibody to visualize cilia. Coinjection of membrane-associated mCherry RNA (Cherry, 100 pg) marks cell boundaries. (**E,H**) Percentage of cells with the indicated cilia position. Representative cells with the anterior (A), middle (M) or posterior (P) position of cilia are indicated in (**C**). Significance was assessed by two-tailed t test comparing the frequencies of cilia positioned posteriorly (green). (**E**) Pk3MO1 to CoMO: *p* = 0.0003, Pk3MO2 to CoMO: *p* < 0.0001. (**H**) Pk3MO1+Pk3 to Pk3MO1: *p* = 0.0002. (**F,I**) Cilia length in GRP cells depleted of Pk3, presented as means +/− s.d. ****p < 0.0001, two-tailed t test. Representative images from three to five experiments are shown, and at least 14 explants were examined per group in each experiment. The effects of Pk3MO1 and Pk3MO2 were evident in approximately 90% of the explants. Co-expression of GFP-Pk3 with Pk3MO1 reduced the frequency of short cilia phenotype to 36% (n = 58). (**E,F,H,I**) Data were collected from 6 to 10 explants per group in three independent experiments.

To further investigate the function of Pk3 by depleting the protein in GRP cells, we designed two anti-sense morpholino oligonucleotides (MOs) with nonoverlapping target sequences. Upon injection into early embryos, both Pk3MO1 and Pk3MO2 efficiently blocked the *in vivo* translation of Pk3-GFP RNA but not FLAG-GFP RNA in co-expression experiments (Fig. 2A). When targeted to GRP cells, both MOs severely impaired the positioning of cilia (Fig. 2B–E). Cilia were positioned posteriorly in 68% of the GRP cells that received control MO (CoMO), but the same position of cilia was observed only in 21% of Pk3MO1- and Pk3MO2-injected cells (Fig. 2E). These data suggest that Pk3 controls proper cilia positioning. Notably, cilia in embryos depleted of Pk3 were significantly shorter (Fig. 2C,D,F), indicating an additional role of Pk3 in ciliogenesis. These effects were partially rescued by coinjected GFP-Pk3 RNA (Fig. 2G–I), further demonstrating specificity.

**Figure 3:**
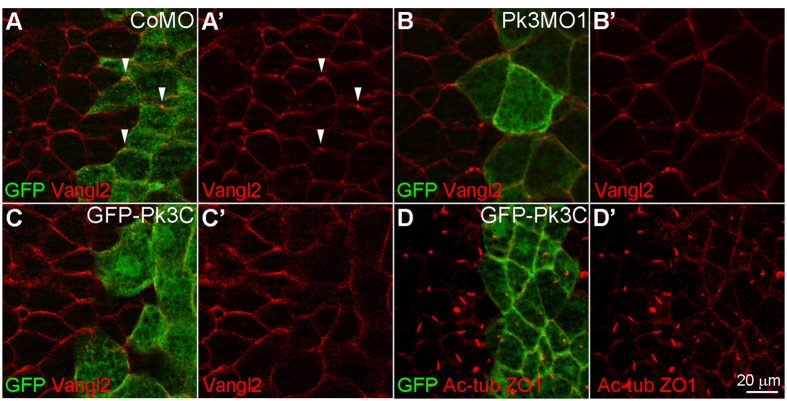
Pk3 is required for the anterior polarization of Vangl2. (**A,B**) Immunostaining of Vangl2 (arrowheads) in stage 15 GRP cells from embryos injected with GFP RNA (0.2 ng, lineage tracer) and CoMO (15 ng, **A,A’**) or Pk3MO1 (15 ng, **B,B’**). (**C,D**) Embryos were injected with 2 ng of GFP-Pk3C RNA, and Vangl2 localization (**C,C’**) and cilia (marked by Ac-tub, **D,D’**) were visualized in GRP cells at stage 15 (**C**) and 17 (**D**) respectively. ZO-1 co-staining reveals cell boundaries. *En face* staining is shown, anterior is to the top. Representative images from three independent experiments are shown, with 6–10 explants per group.

A role for Pk3 in planar polarity was confirmed by the disruption of Vangl2 polarization in Pk3MO1-injected GRP cells but not in CoMO-injected cells (Fig. 3A,B). As Pk3MO2 was less effective and required higher doses, Pk3MO1 was chosen for subsequent analysis. Given that the C-terminus of Pk3 interacts with Vangl2 (Fig. 1F), we suspected that Pk3C expression may have a dominant interfering effect leading to similar PCP defects. Indeed, the asymmetric distribution of Vangl2 was lost in cells expressing GFP-Pk3C (Fig. 3C). In addition, cilia in these cells were short and failed to locate posteriorly, phenocopying Pk3MO injections (Fig. 3D) and suggesting that the C-terminal tail regulates Pk3 activity both in PCP and in ciliogenesis. These effects were not observed after GFP or GFP-Pk3ΔC expression (Supplementary Fig. 1A,B). Of note, the manipulation of Pk3 levels led to occasional alteration of cell size, supporting a possible role of Pk3 in cell cycle regulation (Fig. 3B,C and data not shown). Since these changes were variable and visible only in some embryos, we did not assess this defect in subsequent experiments.

Taken together, our observations demonstrate roles of Pk3 in PCP and cilia development in GRP cells.

### Pk3 is required for pericentriolar material recruitment to the basal body

**Figure 4:**
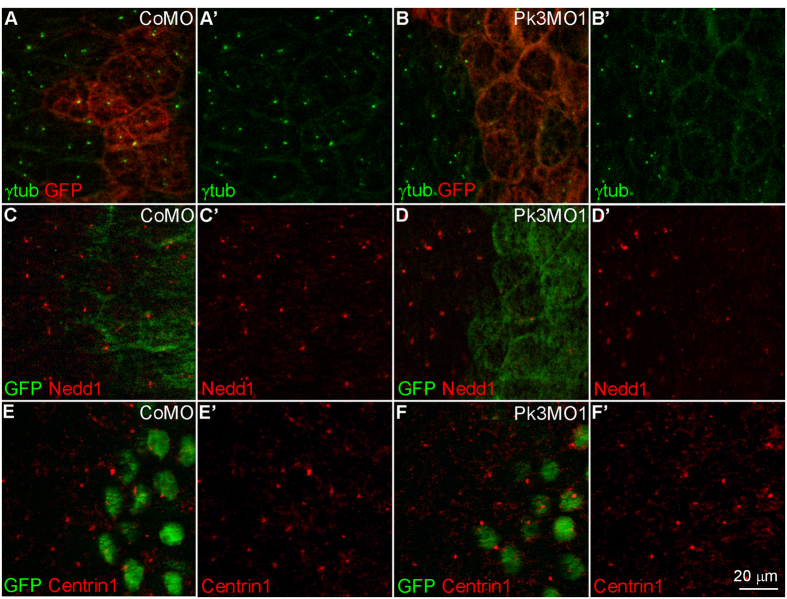
Pk3 depletion prevents γ-tubulin and Nedd1 recruitment to the basal body. Embryos were injected with 0.2 ng of RNA encoding GFP (**A–D**) or Histone-GFP (**E,F**) as a lineage tracer and CoMO (**A,C,E**) or Pk3MO1 (**B,D,F**) (15 ng each). GRP explants from stage 17 embryos were double stained for GFP and γ-tubulin (**A,B**), Nedd1 (**C,D**) or Centrin1 (**E,F**). *En face* staining is shown, anterior is at the top. Representative images from three to five independent experiments are shown, with 6–10 explants examined per group. The effects of Pk3MO1 on γ-tubulin and Nedd1 are detectable in the majority of cells depleted of Pk3 (n > 100).

Given the cilia growth defects in Pk3-depleted cells, we examined centrosomal markers in GRP cells depleted of Pk3. γ-tubulin, a component of the pericentriolar material (PCM) required for microtubule nucleation, colocalized with the centriole marker Centrin1 at the basal body in GRP cells (Supplementary Fig. 2A). We also noticed that γ-tubulin gradually accumulated at the basal body by stage 17 (data not shown). While this localization of γ-tubulin was unaffected in CoMO-injected GRP cells, the staining was strongly reduced in the majority of Pk3-depleted GRP cells (Fig. 4A,B). We next assessed the localization of Nedd1 that is essential for γ-tubulin recruitment to the centrosome^54^,^55^,^56^. Endogenous Nedd1 colocalized with γ-tubulin at the basal body in GRP cells (Supplementary Fig. 2B), and this staining pattern was disrupted by Pk3MO1 but not by CoMO (Fig. 4C,D). By contrast, localization of Centrin1 remained unchanged in cells injected with either Pk3MO1 or CoMO (Fig. 4E,F). These findings suggest that Pk3 regulates basal body organization by modulating the recruitment of γ-tubulin and Nedd1.

### Wtip associates with Pk3 and modulates its localization

**Figure 5:**
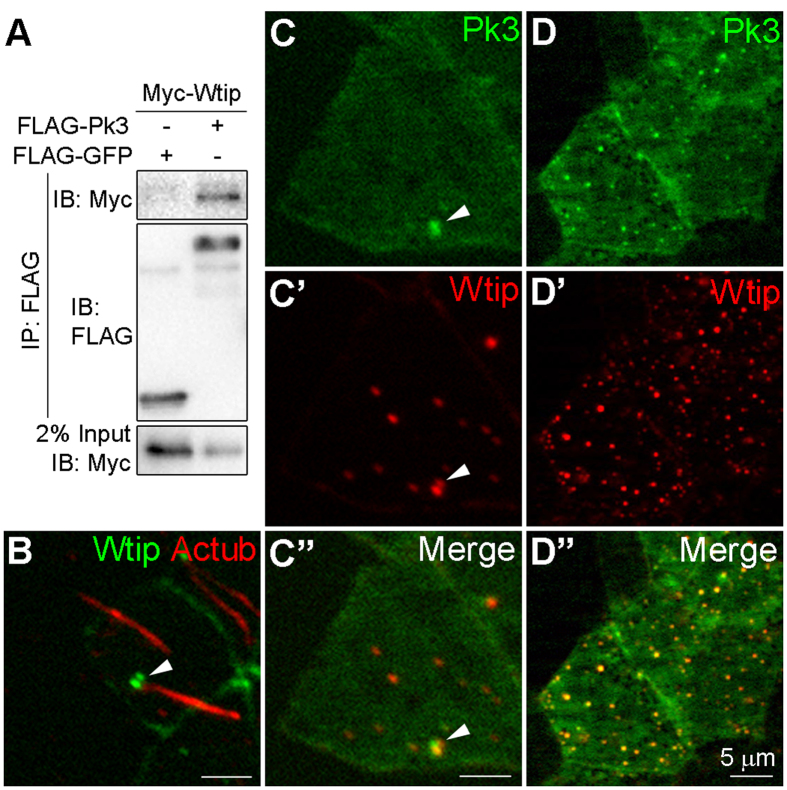
Pk3 and Wtip physically interact and colocalize at the basal body of GRP cells. (**A**) Protein interactions between Wtip and Pk3. HEK293T cells expressing Myc-Wtip and FLAG-Pk3 proteins were lysed and immunoprecipitated with anti-FLAG agarose beads. Protein levels were assessed after immunoblotting with anti-FLAG and anti-Myc antibodies. (**B**) Localization of Wtip at the base of the cilium in GRP cells. Embryos were injected with GFP-Wtip RNA (150 pg). Protein localization is revealed by epifluorescence and staining of acetylated α-tubulin (Ac-tub) in stage 15 GRP explants. (**C,D**) Colocalization of Pk3 and Wtip in the GRP cells. Embryos were injected with 250 pg of GFP-Pk3 RNA plus 100 pg (**C**) or 250 pg (**D**) of HA-RFP-Wtip RNA. Epifluorescence in stage 15 GRP explants is shown.

With the exception of Vangl2, proteins that bind Pk3 and may regulate its functions in PCP and at the basal body are unknown. Wilm’s tumor protein 1-interacting protein (Wtip) is a member of the Ajuba LIM protein family^57^ and plays essential roles in cilia functions and Vangl2-dependent mitotic spindle orientation^58^. Wtip is, therefore, among a few proteins implicated in both PCP and ciliogenesis; however, the biochemical link of Wtip to PCP components has not been established. Since both Wtip and Pk3 contain LIM domains that are capable of dimerization^59^, we hypothesized that the two proteins may physically interact. Supporting this possibility, robust biochemical association of Wtip and Pk3 was observed in pulldown assays using lysates from transfected HEK293 cells (Fig. 5A).

To confirm that Wtip is present in GRP cells during the relevant developmental stages, we carried out whole-mount *in situ* hybridization. Wtip transcripts were only weakly detected in gastrula embryos, but became abundantly expressed in the neural and neural crest tissue of stage 18 neurulae (Supplementary Fig. 3A–E), consistent with a previous study^60^. Both Wtip and Pk3 RNAs were present in GRP explants (Supplementary Fig. 3F-J), supporting the possibility of the interaction between the two proteins in the GRP. We next assessed Wtip distribution in GRP cells. GFP-Wtip was visible as two puncta at the base of the cilium (Fig. 5B), and coexpression of GFP-Pk3 and HA-RFP-Wtip revealed substantial but not identical colocalization of both proteins at the basal body (Fig. 5C–C”). At higher levels, Wtip formed cytoplasmic aggregates that included GFP-Pk3 (Fig. 5D–D”).

These observations demonstrate that Pk3 and Wtip interact *in vitro* and *in vivo* and suggest that Wtip is involved in the recruitment of Pk3 to the basal body in GRP cells.

### Pk3 and Wtip function together in GRP cells

**Figure 6:**
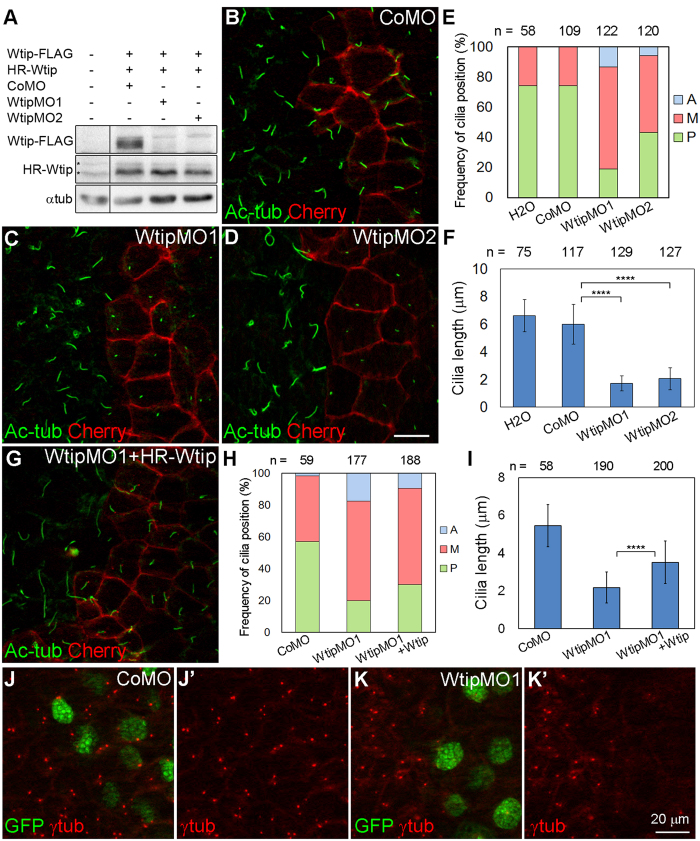
Wtip depletion affects posterior localization and growth of GRP cilia. (**A**) Efficiency and specificity of the Wtip knockdown. Embryos were injected with Wtip-FLAG RNA (1 ng), HA-RFP-Wtip RNA (HR-Wtip, 0.5 ng), control MO (CoMO, 30 ng), WtipMO1 (10 ng), or WtipMO2 (30 ng) as indicated. Embryo lysates obtained at stage 12 were immunoblotted with anti-FLAG and anti-HA antibody. Asterisks mark nonspecific signals from anti-HA antibody. α-tubulin (αtub) is a control for loading. (**B–I**) Effects of Wtip depletion (**B–F**) and rescue (**G–I**) on cilia position and length. Embryos were injected with CoMO (**B**), WtipMO1 (**C**), WtipMO2 (**D**) or WtipMO1 plus HA-RFP-Wtip RNA (75 pg) (**G**). GRP explants were prepared at stage 17 and stained with anti-acetylated α-tubulin (Ac-tub) antibody to visualize cilia. Membrane-associated mCherry marks the boundaries of cells injected with MOs. Anterior is to the top. (**E,H**) Percentage of cells with the indicated cilia position. The position of each cilium was assigned to the A, M or P location in each cell, as in Fig. 2. Significance was assessed by two-tailed t test comparing the frequencies of cilia positioned posteriorly (green). WtipMO1 to CoMO: *p* < 0.0001, WtipMO2 to CoMO: *p* = 0.0009. WtipMO1+ Wtip to WtipMO1: *p* = 0.35. (**F,I**) Bar graph showing the means +/− s.d. of cilia length in GRP cells depleted of Wtip. ****p < 0.0001, two-tailed t test. Representative images from three independent experiments are shown, and at least 7 explants were examined per group. The effects of WtipMO1 and WtipMO2 were visible in over 80% of the explants. Co-expression of HA-RFP-Wtip with WtipMO1 reduced the frequency of short cilia phenotype to 48% (n = 23). (**E,F,H,I**) Data are collected from six embryos in two independent experiments. (**J,K**) Embryos were injected with 100 pg of Histone-GFP RNA and CoMO (**J,J’**) or WtipMO1 (**K,K’**) (10 ng each). GRP explants from stage 17 embryos were double stained for GFP and γ-tubulin. Representative images from three independent experiments are shown, 6–10 explants were examined per group. The effect of WtipMO1 on γ-tubulin staining was visible in approximately 80% of the explants.

To gain insight into the role of Wtip in basal body organization, we interfered with its function using two nonoverlapping MOs (WtipMO1 and WtipMO2), which inhibited *in vivo* translation of Wtip-FLAG RNA carrying MO target sequences but not HA-RFP-Wtip RNA (Fig. 6A). Injection of both MOs caused cilia positioning and growth defects in GRP cells, while CoMO had no effect, confirming specificity (Fig. 6A–D). In contrast to CoMO injections, cilia in Wtip-depleted cells were short and failed to acquire a posterior position (Fig. 6E,F), phenocopying Pk3 depletion. Co-injection of HA-RFP-Wtip partially rescued Wtip knockdown phenotype (Fig. 6G–I). Similar to the effect of Pk3 depletion, we found that γ-tubulin staining at the basal body was reduced in cells injected with WtipMO1 (Fig. 6J,K), demonstrating a possible function of Wtip at the basal body.

**Figure 7:**
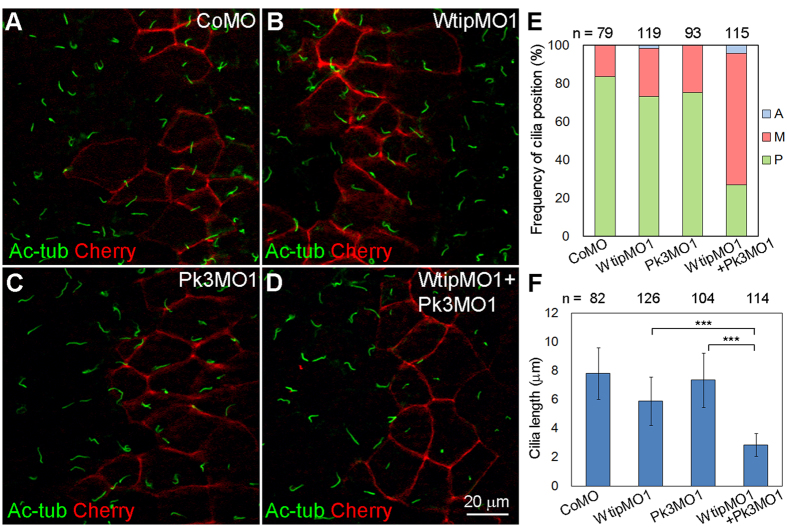
Wtip functionally interacts with Pk3 to regulate GRP cilia growth and positioning. (**A–D**) Synergistic effects of Wtip and Pk3 depletion. Embryos injected with membrane-mCherry (Cherry) RNA and CoMO (5 ng, **A**), WtipMO1 (2.5 ng, **B**), Pk3MO1 (2.5 ng, **C**) or WtipMO1 plus Pk3MO1 (**D**) were stained with anti-acetylated α-tubulin (Ac-tub) antibody to visualize cilia. Membrane-mCherry marks the boundaries of the targeted cells. *En face* staining is shown, anterior is to the top. Representative images from three independent experiments are shown, with at least 14 explants examined per group in each experiment. While WtipMO1 and Pk3MO1 alone caused short cilia in 20% and none of the explants respectively, co-injection of both MOs increased the frequency of short cilia phenotype to 84% (n = 32). (**E**) Percentage of cells with the indicated cilia position. The position of each cilium was assigned to the A, M or P location in each cell. Significance was assessed by one-way ANOVA comparing frequencies of posteriorly positioned cilia (green) in the WtipMO1+Pk3MO1 group and the WtipMO1 group (*p* < 0.001) or the Pk3MO1 group (*p* < 0.001). (**F**) Bar graph showing the means +/− s.d. of cilia length in GRP cells injected with MOs. ***p < 0.001, one-way ANOVA. (**E,F**) Data are collected from six embryos in two independent experiments.

We next decided to investigate whether Wtip and Pk3 function together to regulate PCP and ciliogenesis. When sub-threshold doses of WtipMO1 or Pk3MO1 were separately injected into embryos, the positioning and length of GRP cilia were largely unaffected (Fig. 7A–C). Coinjection of both MOs, however, significantly decreased cilia length and the percentage of cells with posteriorly localized cilia (Fig. 7D–F). These findings indicate that Pk3 and Wtip cooperate to control GRP cilia development.

## Discussion

Our study examined subcellular localization and developmental roles of Pk3, a Prickle family member. Pk3 was enriched at the basal body of GRP cells and was recruited to the anterior cell cortex in the presence of exogenous Vangl2. Besides the involvement of Pk3 in PCP, loss-of-function experiments revealed its novel role in basal body organization and function. We propose that this role may be mediated by Wtip, another LIM domain protein that binds Pk3 and synergizes with Pk3 to regulate ciliogenesis. These findings suggest that, in addition to cell polarity, PCP proteins regulate centrosome/basal body organization.

We hypothesize that the polarization of the exogenous Pk3-Vangl2 complex to anterior boundaries of GRP cells reflects the distribution of endogenous proteins. In support of this hypothesis, we demonstrate anterior enrichment of endogenous Vangl2 in the GRP using a previously characterized antibody^6^. We have also raised Pk3-specific antibodies that recognize the *Xenopus* protein by immunoblot analysis and in immunofluorescence staining but failed to detect endogenous Pk3 due to its low abundance (data not shown). These results are consistent with PCP protein distribution reported for the mouse node and other vertebrate tissues^3^,^4^,^6^,^20^,^22^,^61^. The polarization of GFP-Pk3 is only apparent in the presence of exogenous Vangl2, suggesting that the amount of endogenous Vangl2 is limiting in this assay.

In contrast to our findings, a recent study demonstrated posterior rather than anterior enrichment of GFP-Prickle2 (Pk2) and GFP-Vangl1 in *Xenopus* skin at tailbud stages^39^. This diverse localization could relate to functional differences between Pk3 and Pk2 proteins, as exemplified by the opposite distribution of Prickle^PK^ and Prickle^SPLE^ isoforms in *Drosophila*^62^,^63^. To address this issue, we assessed Pk3 polarity in stage 30 epidermal cells with or without Vangl2 coexpression. Unlike Pk2, Pk3 did not exhibit cortical polarization on its own (Supplementary Fig. 4). In the presence of Vangl2, some cells revealed Pk3 enrichment at the posterior or anterior domain, but Pk3 was uniformly cortical in other cells (Supplementary Fig. 4). Thus, Pk3 polarization requires specific protein structure and is context-dependent (i.e. determined by tissue type and the developmental stage).

Our analysis reveals the critical role of Pk3 C-terminal fragment for its association with Vangl2. Whereas *Drosophila* Pk also binds to Vang via its C-terminus^33^, the C-termini of *Drosophila* Pk and *Xenopus* Pk3 are not conserved (only 10% identity, data not shown), indicating that the secondary or tertiary protein structure may be important for the interaction. Overexpression of Pk3 C-terminus (Pk3C) interfered with Vangl2 localization and posterior cilia positioning, providing further support to the view that formation of the Pk3-Vangl2 complex is essential for PCP. Since Pk3C was unable to polarize on its own or in response to exogenous Vangl2 (data not shown), other domains of Pk3 must be required for its process.

Besides the role of Pk3 in PCP, our findings also revealed a novel function of Pk3 in basal body organization and ciliogenesis. We observed GFP-Pk3 localized at the basal body of GRP cells, consistent with the distribution of Pk3 in mammalian cells^44^. Moreover, depletion of Pk3 led to reduced staining of γ-tubulin and Nedd1 at the basal body without affecting Centrin1 and acetylated α-tubulin, indicating that pericentriolar material is disorganized. Furthermore, Pk3 knockdown inhibited ciliogenesis, possibly related to the abnormal basal body function. Notably, vertebrate Vangl proteins are thought to promote the posterior positioning of nodal cilia without a significant effect on cilia length^19^,^21^,^22^. This evidence implies that Pk3 regulates basal body and ciliogenesis independently of Vangl2. In addition to these effects on basal body organization, Pk3 may regulate microtubule orientation and vesicular trafficking as suggested for other Prickle family proteins^41^,^64^,^65^. Future studies are needed to further define mechanisms that involve Pk3 and regulate ciliogenesis and PCP.

We propose that Pk3 function in basal body organization is mediated by Wtip, a vertebrate-specific LIM-domain-containing protein. Wtip belongs to the Ajuba LIM family that localize to the centrosome and has been implicated in Wnt and Rho GTPase signaling^57^,^66^,^67^. Our hypothesis is supported by several lines of evidence. First, Wtip physically interacts with Pk3 and is able to recruit it to the cytosolic puncta. Second, Wtip knockdown phenocopies Pk3 depletion by affecting both cilia length and γ-tubulin recruitment to the basal body. Third, Wtip synergizes with Pk3 to regulate ciliogenesis in the GRP. Supporting the role of Wtip in basal body organization, zebrafish Wtip morphants exhibit striated rootlet deficiencies^58^. On the other hand, the effect of Wtip depletion on posterior cilia positioning suggests that, similar to Pk3, Wtip is involved in the establishment of PCP. Consistent with this possibility, Wtip binds Ror2, a known Wnt5a receptor implicated in morphogenesis and PCP^68^,^69^,^70^, and synergizes with Vangl2 to regulate spindle orientation in zebrafish embryos^58^,^71^. It remains to be investigated whether Wtip is directly involved in PCP.

At present, the relationship between roles of Pk3 in basal body organization and PCP is unclear. One possibility is the localization of Pk3 at the cell cortex specifies its function in PCP, whereas its enrichment at the basal body is relevant for ciliogenesis. In support of this hypothesis, we observe that Pk3 is recruited to the anterior cortex in the presence of Vangl2, which binds Pk3 and is essential for PCP, whereas Wtip may function to recruit Pk3 to the basal body. The other possibility is that the two functions of Pk3 are interconnected, so that basal body regulation by Pk3 *per se* contributes to PCP. This is in agreement with the proposed roles of the centrosome in directing cell polarity in different systems^72^. Our current data do not allow us to distinguish between the two possibilities.

## Methods

### Plasmids, *in vitro* RNA synthesis and morpholino oligonucleotides (MOs)

Cloning of *Xenopus* Prickle3 (Pk3, GenBank accession number BC154995) into pCS2-FLAG and pcDNA3 has been described^13^. Pk3C (amino acids 373–538) and Pk3∆C (amino acids 1–372) constructs were generated by PCR and subcloned into pXT7-GFP and pCS2-FLAG^73^. FLAG-GFP-Pk3 was made by subcloning GFP-Pk3 into pCS2-FLAG. The full-length coding sequence plus the 5′UTR sequence of Pk3 (JGI Laevis) was obtained by PCR from neurula cDNA and ligated to GFP and pCS107 to produce Pk3-GFP-pCS107. FLAG-GFP was made by subcloning GFP from pEGFP-C1 to pCS2-FLAG. The plasmids encoding the following constructs have been described: GFP-C1^74^, Histone-GFP (a gift of P. Skourides)^75^, membrane-associated mCherry^76^. Mouse HA-Vangl2 plasmid was a gift of Yingzi Yang (Gao *et al*.^70^). *Xenopus* Myc-Wtip was a gift of Greg Longmore^60^. The coding sequence of Wtip was subcloned into pXT7-GFP and pCS105-HA-RFP to generate pXT7-GFP-Wtip, pXT7-GFP-WtipN, pCS2-Wtip-FLAG and pCS105-HA-RFP-Wtip. pXT7-GFP-WtipN contains *Wtip* cDNA fragment corresponding to Wtip amino acids 1–480. Wtip-FLAG-pCS2 encodes Wtip with the C-terminal FLAG epitope. *Xenopus* HA-Vangl2-pCS2 (for immunoprecipitation experiments) was generated by PCR. Details of cloning are available upon request.

Capped mRNAs were synthesized using mMessage mMachine kit (Ambion, Austin, TX). MOs were purchased from Gene Tools (Philomath, OR). The following MOs were used: Pk3MO1, 5′-GGATGCCGCCCGCTCTCTCCCTTA-3′; Pk3MO2, 5′-CTCCTCCTGGAATTACGGAACATCC-3′; control MO (CoMO), 5′-GCTTCAGCTAGTGACACATGCAT-3′^13^, WtipMO1, 5′- TGTCCTCATCGTACTTCTCCATGTC-3′, WtipMO2, 5′-AAGAATCCCTTATGTCACTTGAGCC-3′.

### *Xenopus* embryo culture, microinjections, and *in situ* hybridization

*In vitro* fertilization and culture of *Xenopus laevis* embryos were carried out as previously described^77^. Staging was according to Nieuwkoop and Faber^78^. For microinjections, four-cell embryos were transferred into 3% Ficoll in 0.5x MMR buffer (50 mM NaCl, 1 mM KCl, 1 mM CaCl_2_, 0.5 mM MgCl_2_, 2.5 mM HEPES pH 7.4) and 10 nl of mRNA or MO solution was injected into one or more blastomeres. Amounts of injected mRNA per embryo have been optimized in preliminary dose-response experiments (data not shown) and are indicated in figure legends. *In situ* hybridization was carried out largely as described^79^. The digoxigenin-labeled antisense and sense RNA probes were synthesized *in vitro* with T7 or Sp6 RNA polymerases using Megascript kit (Ambion) from pcDNA3-Pk3 and pXT7-WtipN, respectively.

After *in situ* hybridization, the explants were embedded in cold-water fish gelatin/sucrose mixture and cryosectioned using the Leica cryostat CM3050 at 20 μm as described previously^80^. Images were digitally acquired on a Zeiss Axiophot microscope.

### Immunofluorescence staining

For immunofluorescence staining, stage 17–18 embryos were manually devitellinized and dissected to isolate GRP explants as described by^22^. Epidermal explants were prepared at stage 30. The explants were fixed with MEMFA (0.1 M MOPS, pH 7.4, 2 mM EGTA, 1 mM MgSO_4_ and 3.7% formaldehyde)^79^, MEMFA + 0.1% Triton X-100 for staining of Centrin1^81^, Dent’s fixative (80% methanol +20% DMSO)^82^ for staining of γ-tubulin and Nedd1 or 2% trichloracetic acid (TCA)^83^ for Vangl2 staining. Indirect immunofluorescence staining was performed as described previously^80^, except for Centrin1 staining, the blocking solution was PBS + 0.1% Triton X-100 + 3% BSA + 3% goat serum. The following primary antibodies were used: mouse anti-GFP 1:200 (B-2, Santa Cruz), rabbit anti-GFP 1:400 (A6455, Invitrogen), rabbit anti-HA 1:3000 (Bethyl Labs), mouse anti-acetylated tubulin 1:500 (6-11B-1, Santa Cruz), mouse anti-ZO1 1:200 (Invitrogen), mouse anti-γ-tubulin 1:500 (GTU-88, Sigma), rabbit anti-γ-tubulin 1:500 (ab11321, Abcam), rabbit anti-Vangl2^84^, mouse anti-Nedd1 1:200 (39-J, Santa Cruz), rabbit anti-Centrin1 1:1000 (ab11257, Abcam). Secondary antibodies were Alexa Fluor 488-conjugated (Invitrogen) or Cy3-conjugated 1:300 (Jackson ImmunoResearch). Stained explants were mounted for observation in the Vectashield mounting medium (Vector). Images were captured using a Zeiss AxioImager microscope with the Apotome attachment. Results shown are representative images from 2 to 5 independent experiments with 6–15 explants per group.

### Cell culture and transfection

Human embryonic kidney 293T cells were maintained in DMEM (Corning) with 10% FBS (Gemini) and penicillin/streptomycin (Sigma). Cells growing at 70% confluence were transiently transfected using linear polyethylenimine (M.W. 25,000, Polysciences) as described^85^. Each 35 mm dish of cells received 1.5 μg of pCS2 plasmids encoding FLAG-Pk3, FLAG-GFP, HA-Vangl2 or Myc-Wtip as indicated. For transfection, pCS2 vector DNA was added to plasmid DNA mixture to reach the total DNA amount of 3 μg.

### Immunoprecipitation and Western blot analysis

For immunoprecipitation, cells transfected for 24 hours were lysed in IP buffer (10 mM HEPES pH 7.4, 150 mM NaCl, 1 mM EGTA, 1 mM MgCl_2_, 1% Triton X-100, 1 mM Na_3_VO_4_, 10 mM NaF, 25 mM β-glycerol phosphate), containing protease inhibitor cocktail (cOmplete Mini EDTA-free, Roche). After centrifugation at 16,000 g, the supernatant was incubated with anti-FLAG agarose beads (Sigma) at 4 °C for 1.5 hours. The beads were washed three times with IP buffer, boiled in the SDS-PAGE sample buffer, and subjected to SDS-PAGE and imunoblotting following standard protocols. Western analysis of embryo lysates was carried out essentially as described^86^. Briefly, 5 embryos at stage 10.5 were homogenized in the lysis buffer (50 mM Tris-HCl pH 7.6, 50 mM NaCl, 1 mM EDTA, 1% Triton X-100, 10 mM NaF, 1 mM Na_3_VO_4_, 25 mM β-glycerol phosphate, 1 mM PMSF). After centrifugation at 16,000 g, the supernatant was boiled in the sample buffer and subjected to SDS-PAGE and immunoblotting. The following primary antibodies were used: mouse anti-GFP (B-2, Santa Cruz), mouse anti-FLAG (M2, Sigma), rabbit anti-HA (Bethyl Labs), mouse anti-Myc (9E10). Staining with anti-α-Tubulin antibody (B512, Sigma) was used as loading control. Chemiluminescence was captured by the ChemiDoc MP imager (BioRad).

### Image analysis and quantification

For quantification of GRP cilia length, we used Axiovision software (Zeiss). Ciliated cells were divided equally into anterior, middle and posterior zones. Cilium position was assigned to one of the zones and was scored only when unambiguous. Data were collected from 6–15 explants in 2–3 independent experiments. Two-tailed Student’s t test and one-way ANOVA were performed using Excel (Microsoft) and SigmaPlot (Systat) respectively.

## Additional Information

**How to cite this article**: Chu, C.-W. *et al*. Prickle3 synergizes with Wtip to regulate basal body organization and cilia growth. *Sci. Rep.* **6**, 24104; doi: 10.1038/srep24104 (2016).

## Supplementary Material

Supplementary Information
