# Supplementary material for: Prickle3 synergizes with Wtip to regulate basal body organization and cilia growth
Source: Sci Rep. 2016 Apr 11;6:24104. doi: 10.1038/srep24104 (PMC4827067; doi:10.1038/srep24104)

Supplementary Information:

Prickle3 synergizes with Wtip to regulate basal body organization and cilia growth.

Chih-Wen Chu, Olga Ossipova, Andriani Ioannou and Sergei Y. Sokol*

Supplementary Figure 1.

Pk3 lacking the C-terminal fragment does not affect GRP cilia.

Embryos were injected with 2 ng of RNAs encoding GFP (A, A') or GFP-Pk3ΔC (B, B'). Cilia were visualized by immunostaining of acetylated α-tubulin (Ac-tub) in GRP cells at stage 17. ZO-1 co-staining reveals cell boundaries. *En face* staining is shown, anterior is up.

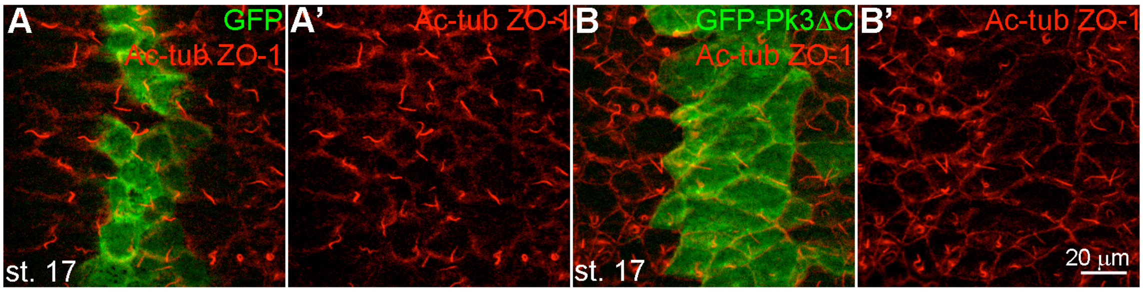

Supplementary Figure 2.

Establishing basal body marker specificity in GRP cells.

GRP explants of stage 17 embryos were co-immunostained for γ-tubulin and Centrin1 (A-A") or Nedd1 (B-B"). Both Centrin1 and Nedd1 colocalize with γ-tubulin at the basal body in the GRP. *En face* staining is shown, anterior is up.

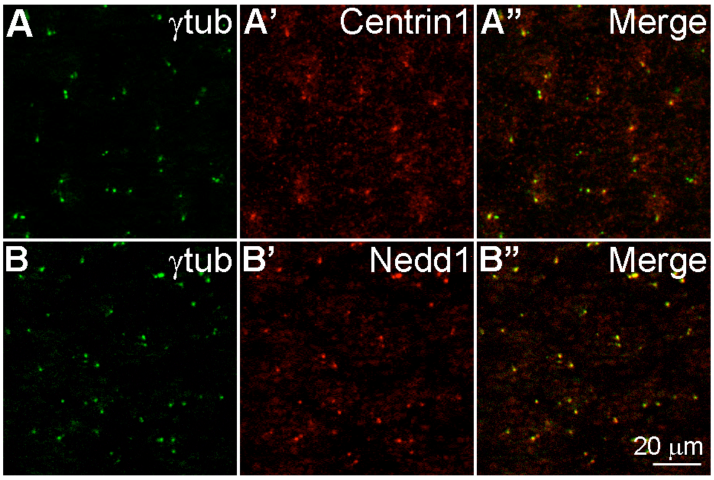

Supplementary Figure 3.

Expression of Wtip and Pk3 in *Xenopus* early embryos.

GRP explants and whole embryos were subjected to wholemount *in situ* hybridization with *Wtip* or Pk3 anti-sense or control sense RNA probes as indicated. (A, B) Stage 10+ gastrulae. *Wtip* transcripts are weakly detected in the animal pole ectoderm when compared to the embryo hybridized with the corresponding sense probe. Animal pole is up. (C-E) Stage 18 neurulae. *Wtip* transcripts are detected in both neural and non-neural ectoderm. The neural folds and the neural crest area are strongly positive. (A-E) Anterior is to the left. (A, B, D) Lateral view, (C, E) dorsal view. (F, G) Both *Pk3* and *Wtip* RNAs are present in the ventral side of GRP explants that were prepared prior to *in situ* hybridization from stage 17/18 embryos. For both anti-sense probes, staining appears stronger at the midline than in the adjacent endoderm. Ventral view is shown, anterior is up. (H-J) Transverse sections of the explants shown in (F, G). Wtip RNA is predominantly in the neural tissue, with lower levels in the GRP (arrow) and the mesoderm (I). Pk3 transcripts are present at the GRP midline (arrow), epidermis and the superficial layer of the neural plate (J). Notochord, N, is indicated by dashed outline.

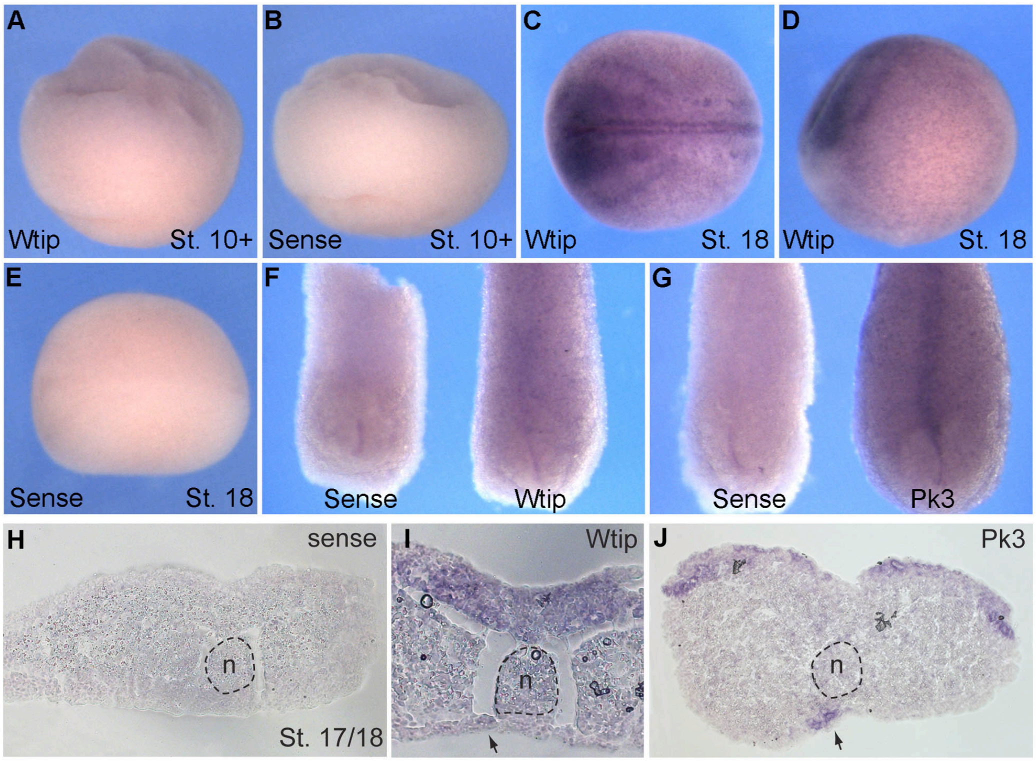

Supplementary Figure 4.

Pk3 becomes polarized in the epidermis in the presence of Vangl2.

Embryos were injected with 100 pg of pCS2-FLAG-GFP-Pk3 DNA (A, B) or 150 pg of GFP-Pk3 RNA plus 150 pg of HA-Vangl2 (VL2) RNA (C-F). Pk3 polarity was visualized by GFP fluorescence in the epidermis of fixed stage 30 embryos. In the absence of HA-Vangl2, fluorescence of injected GFP-Pk3 RNA is not visible at this stage (data not shown). No cortical Pk3 polarization is detectable in representative non-ciliated (A) or ciliated (B) cells, but Pk3 was associated with basal bodies (B). (C-F) Examples of Pk3 polarization in the presence of Vangl2 in ciliated (C, D) and non-ciliated (E, F) cells. Cell borders are marked by co-injected membrane-associated mCherry RNA (Cherry, 100 pg). Asterisks mark the cells showing Pk3 polarization. The anterior-posterior (A-P) and the dorsal-ventral (D-V) axes indicated in E also refer to other panels. Six embryos from two independent experiments were examined. Pk3 was enriched posteriorly in 62 % of mosaically-expressing cells and anteriorly in 38 % of the cells (n = 34).

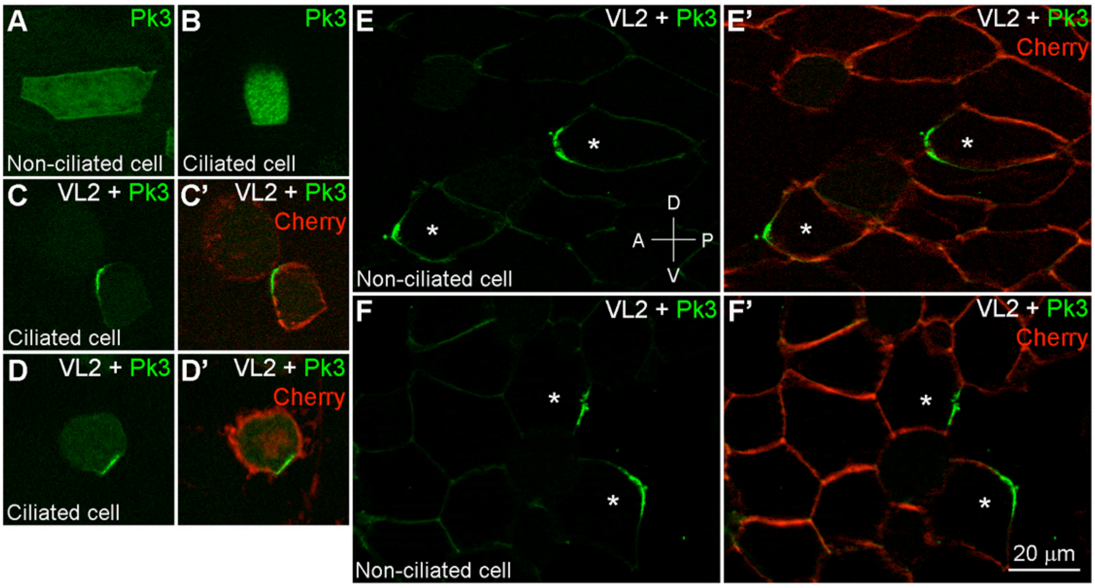

Supplement: Supplementary Information [file srep24104-s1.pdf]
